# MiR-33a targets FOSL1 and EN2 as a clinical prognostic marker for sarcopenia by glioma

**DOI:** 10.3389/fgene.2022.953580

**Published:** 2022-08-17

**Authors:** Wei Wang, Wei Liu, Jing Xu, Hongze Jin

**Affiliations:** Department of Neurosurgery, Changxing People’s Hospital, Changxing, Zhejiang, China

**Keywords:** FOSL1, EN2, mir-33a, glioma, sarcopenia, muscle, systemic homeostasis-related genes

## Abstract

To determine the relationship between glioma and muscle aging and to predict prognosis by screening for co-expressed genes, this study examined the relationship between glioma and sarcopenia. The study identified eight co-downregulated miRNAs, three co-upregulated miRNAs, and seven genes associated with overall glioma survival, namely, KRAS, IFNB1, ALCAM, ERBB2, STAT3, FOSL1, and EN2. With a multi-factor Cox regression model incorporating FOSL1 and EN2, we obtained ROC curves of 0.702 and 0.709, respectively, suggesting that glioma prognosis can be predicted by FOSL1 and EN2, which are differentially expressed in both cancer and aged muscle. FOSL1 and EN2 were analyzed using Gene Set Enrichment Analysis to identify possible functional pathways. RT-qPCR and a dual-luciferase reporter gene system verified that hsa-miR-33a targets FOSL1 and EN2. We found that hsa-mir-33a co-targeting FOSL1 and EN2 has a good predictive value for glioblastoma and skeletal muscle reduction.

## Introduction

Gliomas are the most common primary tumors of the central nervous system (CNS), arising from glial cells or supporting cells (Davis, 2018; Ostrom et al., 2018). The World Health Organization updated their classification system for gliomas in May 2016, taking advantage of advances in molecular genetics and epigenetic research. The revised guidelines aim to allow oncologists to better diagnose, predict treatment outcomes, and improve individualized treatment plans for patients (Louis et al., 2016; Wesseling and Capper, 2018). About 49% of malignant tumors of the CNS are gliomas, which can be found anywhere in the CNS but are most frequently in the frontal and temporal lobes (Schmoldt et al., 1975; Aldape et al., 2015; Ostrom et al., 2018; Miller et al., 2021). Early symptoms of glioma, similar to other benign neurological disorders, include headache, vomiting, loss of vision, seizures, weakness, confusion, and other signs of increased intracranial pressure (Rasmussen et al., 2017; Ozawa et al., 2019). The most common treatment for glioma is surgical resection followed by radiotherapy and chemotherapy (Wen et al., 2020). However, the blood–brain barrier prevents most drugs from reaching the tumor site, so even with combination therapy, the overall survival rate of patients with gliomas is still low (Stummer et al., 2012; Kim et al., 2019; Tan et al., 2020; Miller et al., 2021). Therefore, for early diagnosis and risk assessment of glioma, transcriptomic and epigenomic screening of neuroblastoma-related biomarkers is critical to facilitate early targeted interventions to improve survival.

Sarcopenia is an age-related syndrome of progressive decline in skeletal muscle mass and function, associated with adverse outcomes such as decreased body function, impaired quality of life, physical disability, and death (Cruz-Jentoft et al., 2019). In patients with chronic diseases, malnutrition, and malignancies, sarcopenia is associated with poor prognosis, increasing the risk of recurrence and death (Xia et al., 2020; Williams et al., 2021). Previous studies suggested that sarcopenia and loss of temporal muscle thickness may be associated with lower overall survival (OS) in glioma patients (An et al., 2021; Guven et al., 2021; Huq et al., 2021). There are, however, relatively few studies investigating the association between glioma prognosis and sarcopenia expression profiles.

Typically, sarcopenia is characterized by muscle aging and loss of function and mass in the elderly (Larsson et al., 2019). Muscle loss and sarcopenia are regulated by microRNAs (miRNAs) (Wang et al., 2020; Javanmardifard et al., 2021). MicroRNAs) are endogenous, small RNAs that have a variety of regulatory functions in living organisms (Sun et al., 2017; Chen et al., 2019; Hill and Tran, 2021; Chen et al., 2022). Glioma miRNAs are known to be potential diagnostic markers (Zhou et al., 2018). In previous studies, miRNAs were differentially expressed in the skeletal muscles of elderly people (Larsson et al., 2019). MiRNA-1245a, for instance, has been identified as a potential key molecule for treating glioma-related sarcopenia (An and Wang, 2021). The regulation of miRNAs in the skeletal muscle is influenced by systemic homeostasis (Kim et al., 2020; Podkalicka et al., 2022). The development of sarcopenia and poor outcome for glioma patients are likely to occur as a result of gliomas deteriorating the body’s microenvironmental homeostasis (Magnus et al., 2014; Chen and Kang, 2015). Gliomas may cause sarcopenia by affecting miRNA expression in skeletal muscle.

This study examined miRNAs associated with glioma and sarcopenia and their potentially regulated mRNAs. We screened for co-expressed genes to determine the relationship between glioma and sarcopenia and predict prognosis.

## Methods

### Data collection and processing

The Gene Expression Omnibus (GEO) database provided glioma-derived exosome miRNA transcript datasets (GSE122488), which included 16 standard samples and 22 glioma samples. The miRNAs that did not appear in more than 10 samples were removed. Additionally, 17 old Basal samples and 19 young Basal samples were obtained from skeletal muscle-derived miRNA transcript datasets (GSE23527). For 143 glioma samples, transcriptomic FPKM expression data were obtained from the UCSC Xena website (https://xenabrowser.net/). Expression data were transformed into log_2_ (x+1) when multiple probes corresponded to the same gene.

### Differential expression analysis

GSE122488 and GSE23527 datasets were screened for differentially expressed miRNAs associated with GBM and aging, respectively. Differentially expressed miRNAs were those with *p-*values less than 0.05. Differentially expressed miRNA volcanoes were mapped using the ggplot2 package (Version 3.3.5) and the ggrepel package (Version 0.9.1), and differentially expressed miRNA heatmaps were mapped using the pheatmap package (Version 1.0.12).

### MiRNA target gene prediction and regulatory network mapping

The miRTarBase database contains a large number of experimentally validated miRNA target gene regulatory pairs, ensuring the reliability of the data (Huang et al., 2019). Based on miRTarBase, co-differentially expressed miRNA target genes were examined, and pairs with at least two validated results in Western blot, qPCR, or CLIPseq were retained. Cytoscape (Version 3.8.0) was used to map the miRNA-target regulatory network.

### Gene Ontology and Kyoto Encyclopedia of Genes and Genomes enrichment analyses

For all target genes, the clusterProfiler (Version 4.0.5) package and org. Hs.eg.db (Version 3.13.0) package were used to perform GO and KEGG enrichment analyses, retaining all enriched entries with FDR <0.05 as in previous research studies (Fabris et al., 2020; Huang et al., 2021; Li et al., 2022a; Zhang et al., 2022a; Zhang et al., 2022b; Wang et al., 2022).

### Survival risk prediction model

Training (70%, *n* = 100) and validation (30%, *n* = 43) sets of The Cancer Genome Atlas (TCGA) glioblastoma dataset were randomly divided. Based on the training set samples, a survival risk prediction model was constructed and its performance was then validated using the validation set. To identify the miRNA targets associated with overall survival, a univariate Cox proportional risk regression analysis was conducted on all target genes of common differential miRNAs. The Least absolute shrinkage and selection operator (LASSO) Cox model in the glmnet package (Version 4.1-3) was further used to screen genes with one-way Cox *p* < 0.05 and multicollinearity between the genes was removed. The gene screened by the LASSO regression analysis was then used to construct a multifactorial Cox proportional risk regression model based on the survival package (version 3.2-13) and the survminer package (version 0.4.9). The risk score for each patient was calculated by entering the expression values of the characteristic genes screened by multifactorial Cox regression analysis into the formula. Patients in the training set were segregated into low- and high-risk groups, the relationship between risk score levels was analyzed, and Kaplan–Meier curves were used for prognosis. In addition, the survivalROC (Version 1.0.3) package was used to evaluate the time-dependent subject operating characteristic curve (ROC) of the prediction model over multiple years.

### Gene set enrichment analysis

TCGA-glioma samples were classified into high-expression and low-expression groups by median gene expression values for the signature genes screened. The Gene Set Enrichment Analysis (GSEA) was performed on the Kyoto Encyclopedia of Genes and Genomes (KEGG) pathway using the clusterProfiler package (Version 4.0.5), with the nPermSimple parameter set to 10,000.

### Cell cultivation

The human glioma cell lines U251 and 293T cells were purchased from the Institute of Cell Research, Chinese Academy of Sciences, Shanghai, and cultured in a constant humidity incubator with 5% CO2 in DMEM+10% FBS+1% double-antibody.

### RNA extraction and real-time quantitative PCR

The total RNA was extracted using Trizol (Beyotime, Shanghai, China) according to the instructions and reverse-transcribed into cDNA using the GoScript ™ Reverse Transcription Kit (Promega, Wisconsin, United States), followed by TB Green Premix Ex Taq (Takara, Japan) to determine the mRNA expression levels. The mRNA expression levels were calculated as 2-ΔΔCt, and GAPDH was used as an internal reference.

### Dual-luciferase reporter assay

The pmirGLO-FOSL1-3′ UTR-WT and pmirGLO-EN2-3′ UTR-WT vector plasmids were synthesized by Shanghai Biotech, and the corresponding pmirGLO-FOSL1-3′ UTR-MUT and pmirGLO-EN2-3′ UTR-MUT were used as controls. A total of 500 ng of vector plasmid and 100 pmol of hsa-miR-33a mimics were transfected into 293 T cells, and the fluorescence was detected by using a Dual-Luciferase Assay Kit from Promega (United States) after 24 h.

### Statistical analysis

All data from at least three independent experiments are expressed as mean ± standard deviation. A *t*-test was used to analyze the statistical differences between two independent samples. *p* < 0.05 was considered to be statistically significant.

## Results

### Differentially expressed miRNAs in glioma and muscle

**FIGURE 1:**
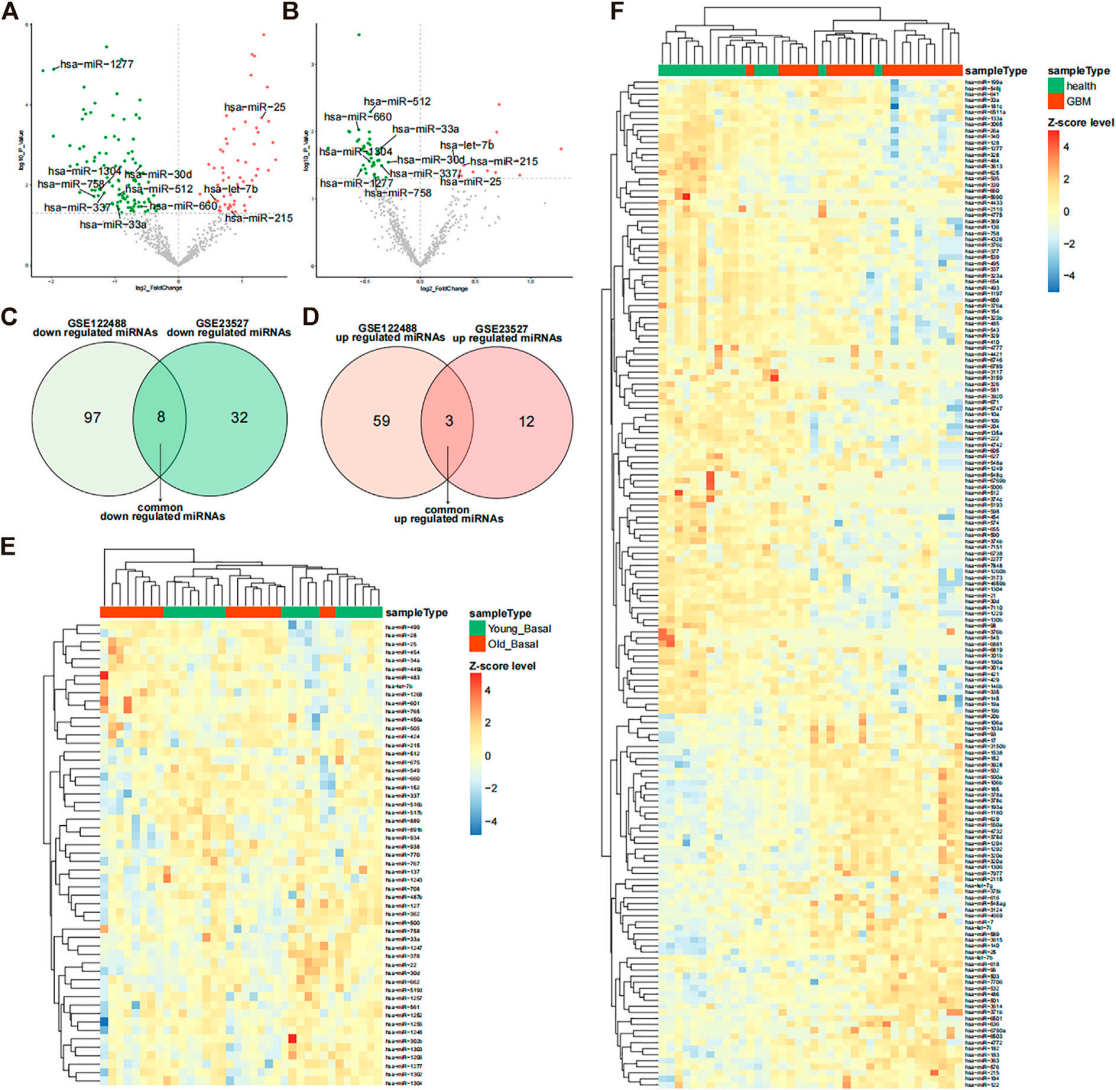
Differentially expressed miRNAs in glioma and muscle. **(A)** Volcano plot of differentially expressed miRNAs in the GSE122488 dataset. Green dots indicate downregulated miRNAs and red dots indicate upregulated differential miRNAs. Common differential miRNAs of both datasets are marked on the plot. **(B)** Volcano plot of differentially expressed miRNAs in the GSE23527 dataset (glioma vs. healthy). Green dots indicate downregulated miRNAs and red dots indicate upregulated differential miRNAs. Common differential miRNAs of both datasets are marked on the plot. **(C,D)** Venn diagram of differential downregulated and upregulated miRNAs in the two datasets. **(E)** Expression heatmap of differentially expressed miRNAs in the GSE23527 dataset. MiRNA expression values were Z-score transformed. **(F)** Expression heatmap of differentially expressed miRNAs in the GSE122488 dataset; miRNA expression values were transformed by the Z-score.

The GSE122488 dataset was screened using differential expression analysis to identify 105 downregulated miRNAs and 62 upregulated miRNAs in glioma (Figure 1A). Aged muscle GSE23527 was also screened for 40 downregulated and 15 upregulated miRNAs (Figure 1B). Furthermore, we obtained eight co-downregulated miRNAs (hsa-miR-512, hsa-miR-660, hsa-miR-1304, hsa-miR-30d, hsa-miR-33a, hsa-miR-337, hsa-miR-1277, and hsa-miR-758) and three co-upregulated miRNAs (hsa-miR-25, hsa-let-7b, and hsa-miR-215) (Figures 1C,D). Different subgroups of the samples displayed different expression patterns of differentially expressed miRNAs, as demonstrated by the expression heatmap (Figures 1E,F). The development of glioma with muscle aging may be closely related to those miRNAs.

### Analysis of common differential miRNA target genes and their functional enrichment

**FIGURE 2:**
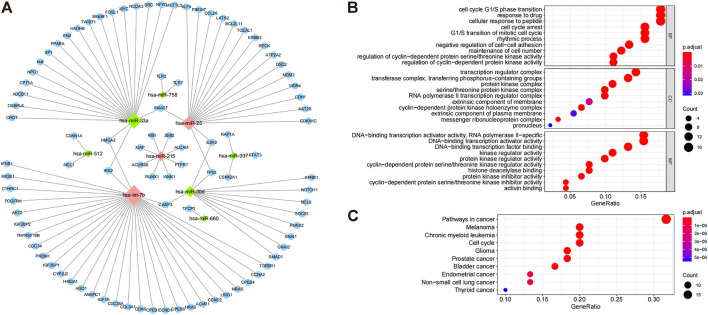
Target gene prediction of common differential miRNAs and their functional enrichment analysis. **(A)** Differential regulation of miRNAs. The diamond-shaped dots indicate miRNAs. Green and red dots indicate downregulation and upregulation, respectively, and blue dots indicate miRNA regulatory targets. **(B)** GO enrichment results of miRNA target genes, highlighting the top 10 GO terms of BP, CC, and MF. **(C)** KEGG enrichment results of miRNA target genes show the top 10 enrichment pathways.

To identify co-differentially expressed miRNAs, we used the miRTarBase database to predict the target genes and screened for miRNA-gene pairs with at least two validation records in a reporter assay, Western blot, qPCR, and CLIPseq. The results showed that miR-1277 and miR-1304 had no target genes; miR-758 and miR512 both had two target genes; miR-660 had one target gene; miR-337 had three target genes; miR-215 had eight target genes; miR-30d had 11 target genes; miR-25 had 18 target genes; miR-33a had 21 target genes; and let-7b had 32 target genes (Figure 2A). The miRNA–target interaction network showed that HMGA2, IRS2, CASP3, TP53, EZH2, and SMAD7 were regulated by two miRNAs, and there was no common target gene between most of the miRNAs (Figure 2A). To further investigate the biological processes affected by these differential miRNAs (hsa-let7b, hsa-miR-25, hsa-miR-30d, hsa-miR-33a, hsa-miR-337, hsa-miR-512, hsa-miR-660, and hsa-miR-758), GO and KEGG enrichment analyses were performed for all common differential miRNAs, involving 1,173 GO pathways (including 1083 BP, 12 CC, and 78 MF) and 35 KEGG pathways. The enrichment results indicated that these miRNAs might be involved in the cell cycle, gene transcription, and cancer-related pathways (Figures 2B,C). Accordingly, these miRNAs may play an essential role in cancer development by targeting specific mRNAs.

### Prognostic value of miRNA target genes

**FIGURE 3:**
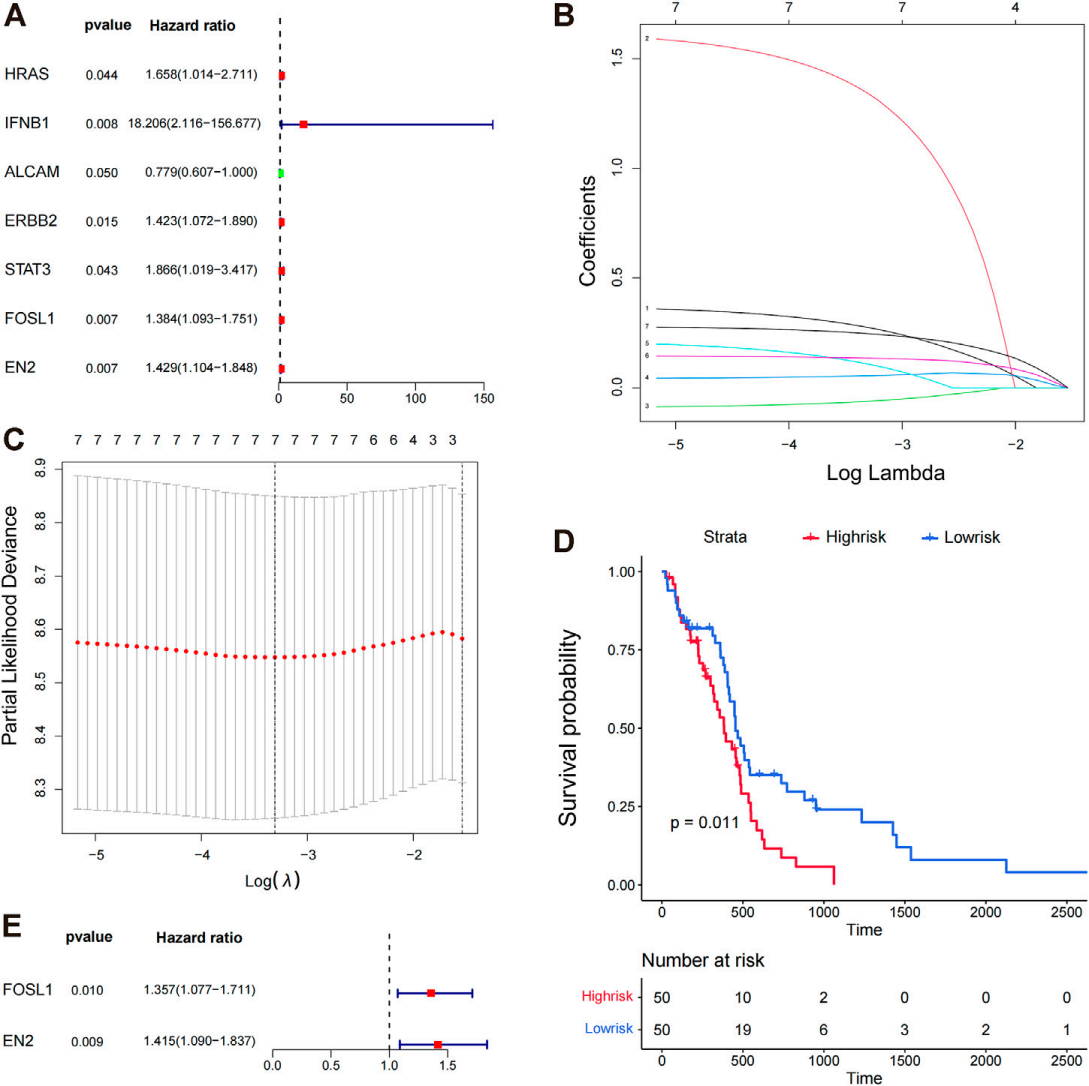
Prognostic value of miRNA target genes. **(A)** Genes targeted by significant miRNAs were analyzed by univariate Cox proportional risk regression. **(B,C)** Lasso regression analysis was conducted on the seven target genes screened by univariate Cox proportional risk regression analysis, and the seven best genes were screened again by Lasso regression. **(D)** K–M plots show the survival differences between high- and low-risk groups in the training set samples. **(E)** Survival risk regression model was constructed using multi-factor Cox proportional risk regression and stepwise regression, and two genes were retained, FOSL1 and EN2.

TCGA training set samples were used to construct a model of glioma-related survival risk prediction. KRAS, IFNB1, ALCAM, ERBB2, STAT3, FOSL1, and EN2 were screened by univariate Cox proportional risk regression (Figure 3A). ALCAM was a protective factor for patient survival, whereas the others were risk factors. The Cox regression analysis revealed an association between high IFNB1 expression and poor prognosis. We then used LASSO Cox regression to further remove the multicollinearity between genes, and LASSO regression did not reject any genes (Figure 3C). Finally, we obtained a convergent patient survival risk regression model using multifactorial Cox proportional risk regression and stepwise regression with two characteristic genes, FOSL1 (hazard ratio, 1.357; 95% CI, 1.077 to 1.711; *p* = 0.010), and EN2 (hazard ratio, 1.415; 95% CI, 1.090 to 1.837; *p* = 0.009) (Figure 3E). The formula for predicting the survival risk is risk score = (FOSL1 expression × 0.3056) + (EN2 expression × 0.3471). FOSL1 and EN2 may affect the prognosis of gliomas independently. The Kaplan–Meier survival analysis was used to compare the survival differences between the high- and low-risk groups based on the median values of the risk scores of the training set samples. The high-risk group’s prognosis was worse than the low-risk group’s *(p* = 0.011) (Figure 3D). Therefore, the multifactorial Cox model developed by FOSL1 and EN2 can better predict glioma prognoses.

### Prognostic model construction for glioma

**FIGURE 4:**
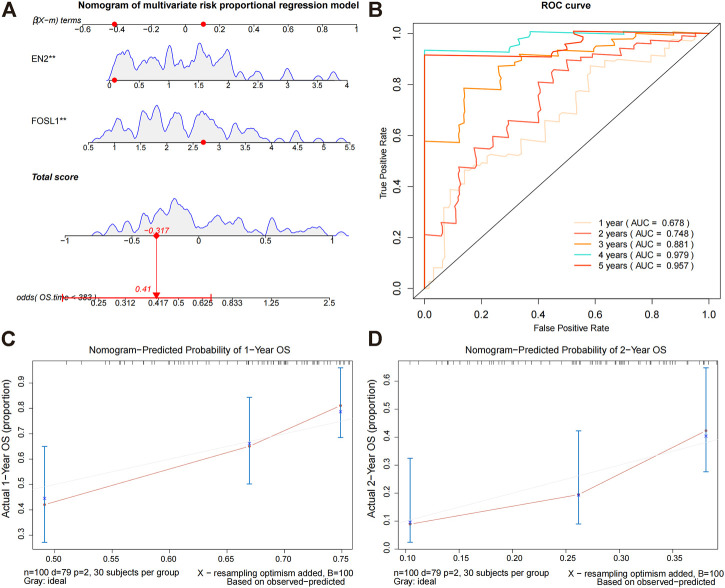
Constructing a nomogram prediction model. **(A)** Nomogram prediction model for prognostic risk. **(B)** Time-dependent ROC curves for the overall survival of the training set. During the first, second, third, and fourth years, AUC values were evaluated. **(C,D)** Calibration curves for predicting the overall survival of the training set at 1 and 2 years.

Based on the prediction model, we constructed a nomogram (Figure 4A) and plotted the calibration curves for the model predicting overall survival at 1 and 2 years (Figures 4C,D). To evaluate the model’s predictive performance, we plotted time-dependent ROC curves. AUCs for the training sets 1, 2, 3, 4, and 5 years were 0.678, 0.748, 0.881, 0.979, and 0.957, respectively (Figure 4B). These results suggest that this nomogram model has good predictive power.

### Nomogram prediction model evaluation

**FIGURE 5:**
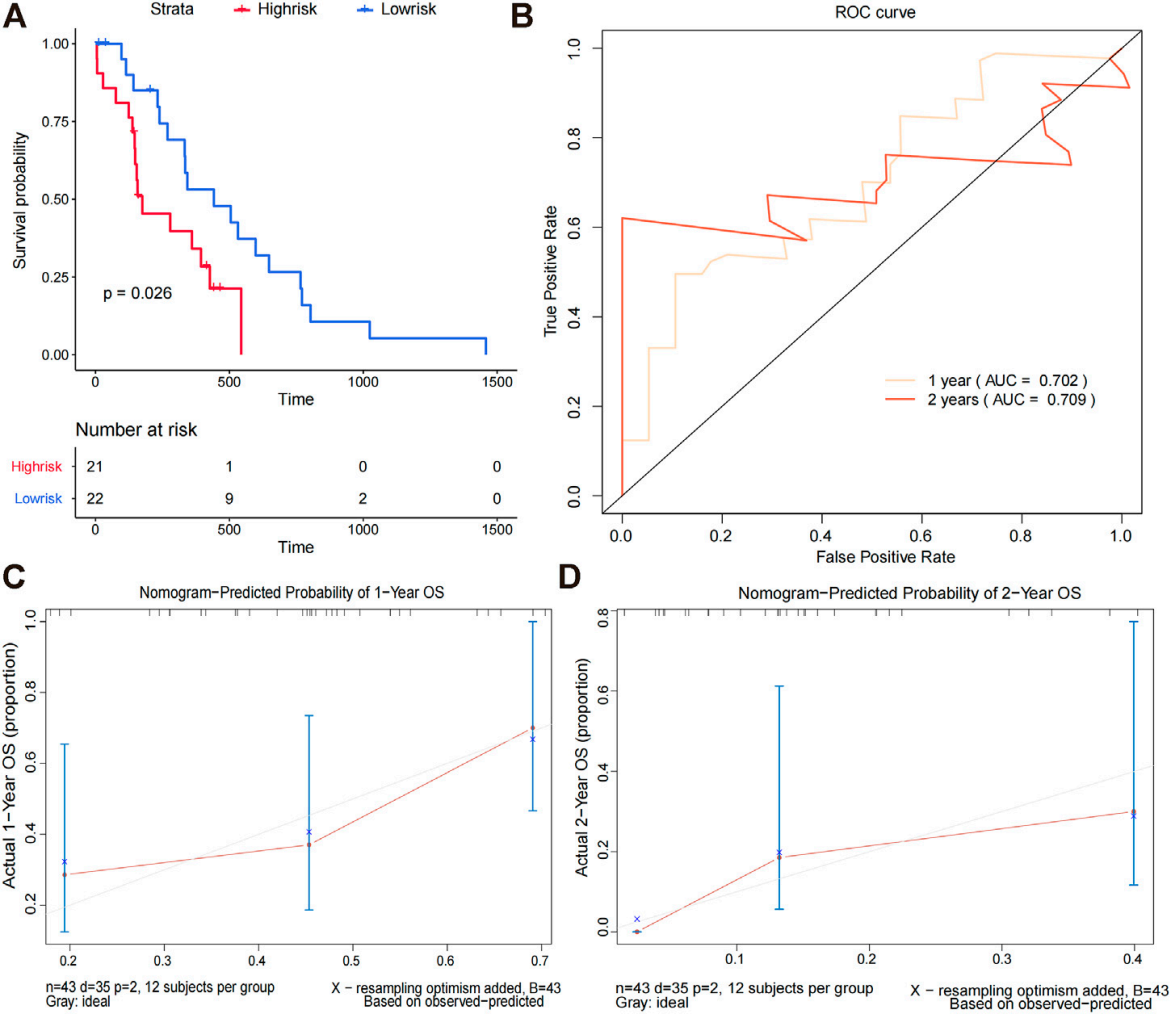
Nomogram prediction model evaluation. **(A)** Difference in the overall survival between high-risk and low-risk groups. **(B)**We evaluated the AUC values at 1 and 2 years based on time-dependent ROC curves for the overall survival of the validation set. **(C,D)** Calibration curves for predicting the overall survival in the validation set at 1 and 2 years.

**FIGURE 6:**
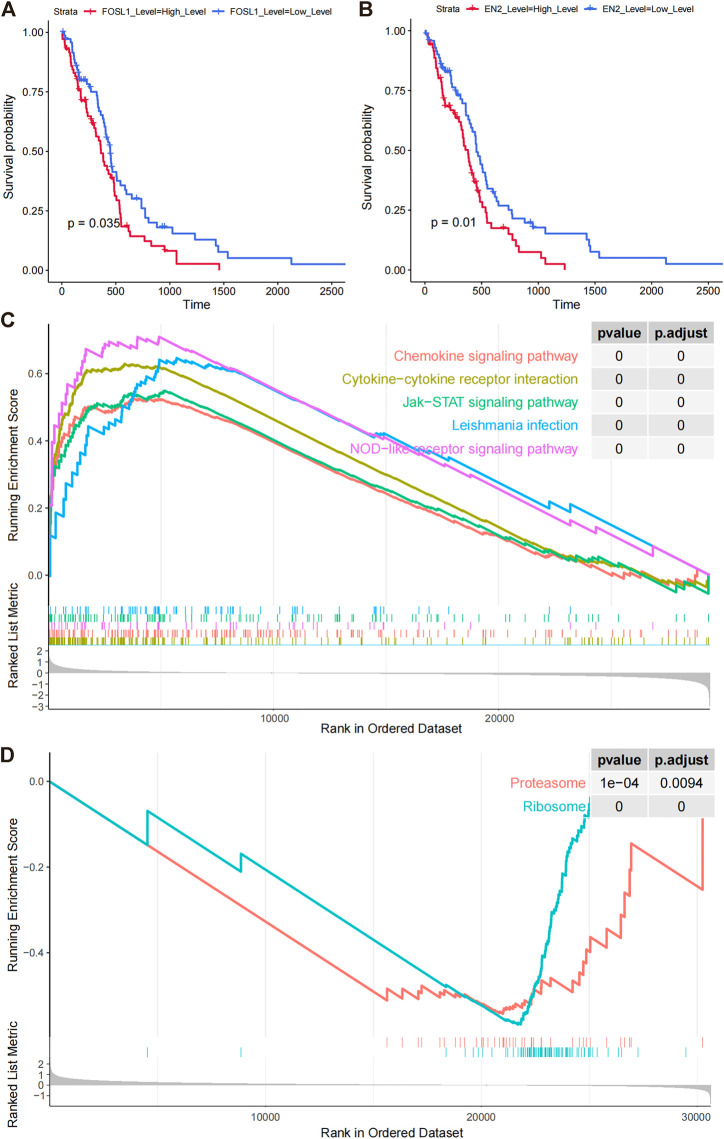
Prognostic model signature genes analyzed by GSEA. **(A,B)** Overall survival differences among FOSL1 and EN2 high- and low-expression groups (grouped by median expression values). **(C)** GSEA enrichment of KEGG pathways in FOSL1 high- and low-groups. **(D)** GSEA significantly enriched the KEGG pathway in the high- and low-EN2 expression groups.

We calculated the survival scores of the validation set samples and compared the survival differences between the high-risk and low-risk groups to further validate the performance of the survival risk prediction model. The high-risk group had a poor prognosis in the validation set (Figure 5A). Additionally, we plotted time-dependent ROC curves of the risk model to predict overall survival at 1 and 2 years for the validation set samples with AUC values of 0.702 and 0.709, respectively (Figure 5B). To investigate whether FOSL1 and EN2 are independent prognostic factors, we divided all TCGA-glioma samples into high- and low-expression groups based on median gene expression values. The Kaplan–Meier survival analysis was then used to compare the survival differences between the high- and low-expression groups. The results showed that both the high-expression groups of FOSL1 and EN2 had poor prognosis (Figures 6A,B).

### Gene expression analysis of prognostic models

In comparing high- and low-expression groups of FOSL1 and EN2, we examined the functional pathways involved with these proteins. FOSL1-related GSEA analysis significantly enriched 23 KEGG pathways, including 22 activated pathways (NES >0), such as leukocyte transendothelial migration, VEGF signaling pathway, and apoptotic cell death. FOSL1-associated top five pathways were plotted using GSEA (Figure 6C). Based on the EN2-related GSEA analysis, only two functional pathways were enriched, proteasome and ribosome (Figure 6D).

### Hsa-miR-33a targets and regulates the expression of *FOSL1* and EN2

**FIGURE 7:**
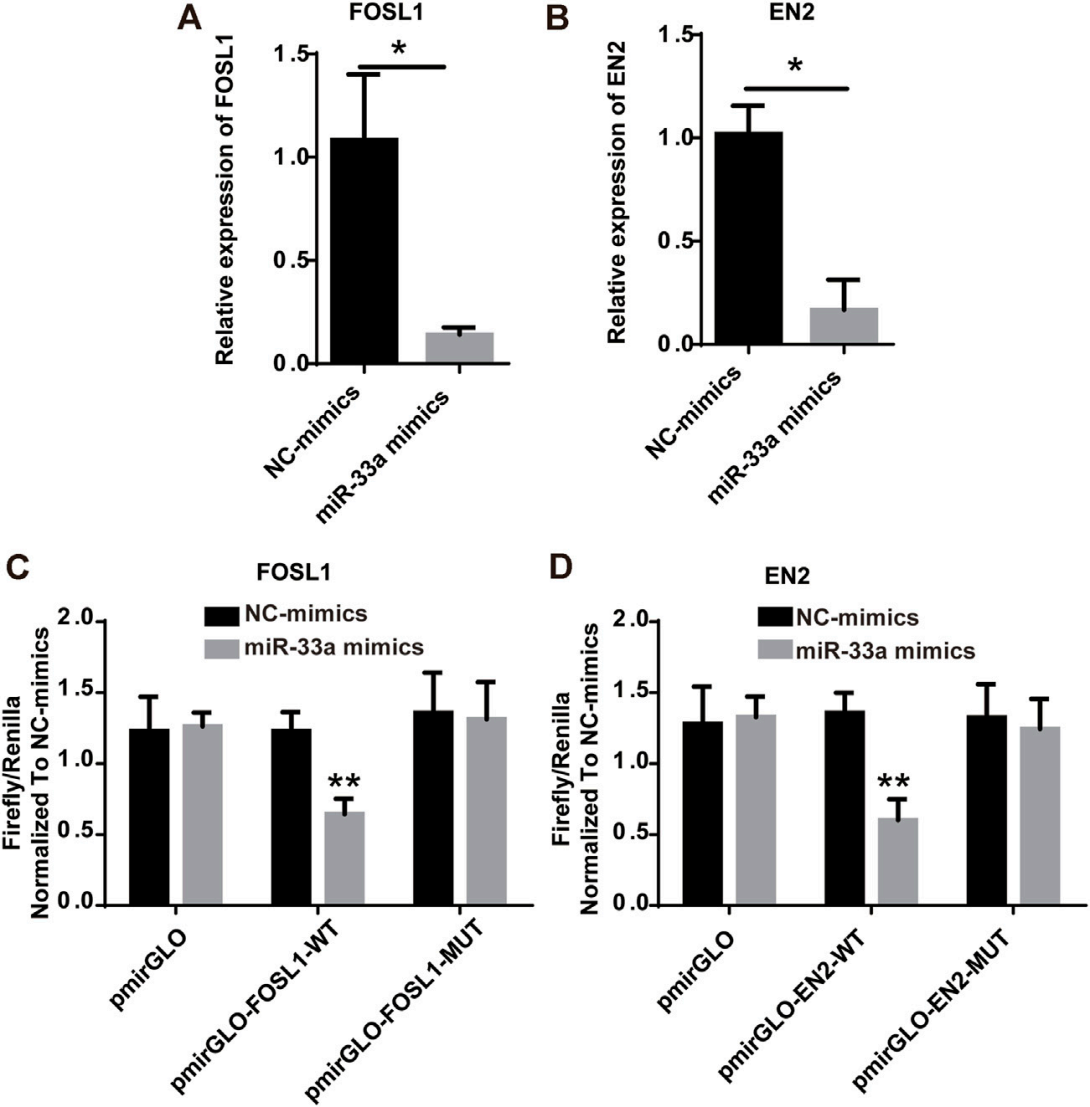
Hsa-miR-33a targets and regulates the expression of FOSL1 and EN2. **(A)** RT-qPCR results indicate that FOSL1 expression is lower in miR-33a-treated glioma cell lines than in the control. **(B)** RT-qPCR results indicated that EN2 expression was lower in miR-33a-treated glioma cell lines than in the control. **(C)** Dual-luciferase reporter gene system indicates the targeting relationship between FOSL1 and hsa-miR-33a. **(D)** Dual-luciferase reporter gene system indicates the targeting relationship between EN2 and hsa-miR-33a. *- *p* < 0.05 and **- *p* < 0.01.

FOSL1 and EN2, which are target mRNAs of hsa-miR-33a, can be used to predict the prognosis of glioma based on the results of the bioinformatics analysis. As risk factors for glioma, both FOSL1 and EN2 can be considered and further verified to be regulated and targeted by hsa-miR-33a. Glioma cell lines were treated with mimics of hsa-miR-33a. As shown in Figures 7A,B RT-qPCR analysis of mimics-treated glioma cell lines showed decreased expression of FOSL1 and EN2. We then used the dual-luciferase reporter gene system to detect the regulation of miRNAs and target genes (Figure 7C,D). The results indicate that miR-33a targets and regulates FOSL1 and EN2. In summary, miR-33a with differential expression in glioma and sarcopenia may affect the prognosis of glioma by targeting and regulating FOSL1 and EN2.

## Discussion

We identified co-expressed differential miRNA genes in glioma and skeletal muscle miRNA datasets. Target genes were predicted using the miRTarBase database after functional annotation according to GO and KEGG. ROC curves were plotted to evaluate the prognostic value of the aforementioned target genes in glioma. Multivariate Cox regression was used to construct a model of prognosis prediction for glioma. The model was evaluated by plotting ROC curves and K–M curves. This model accurately predicted the prognosis for glioblastoma based on correlation analysis, providing an exciting pathway for clinical applications.

Glioma is the most common type of malignant tumor in the central nervous system, and its pathogenesis is complex, involving genetic background, gene mutation, tumor microenvironment, and other aspects (McKinnon et al., 2021). However, most GBMs have no identifiable risk factors for tumor development. Some rare familial cancer syndromes such as Lynch syndrome, neurofibromatosis type 1, and tuberous sclerosis have increased the risk of developing glioma (Ostrom et al., 2014; Wen et al., 2020). Glioma is characterized by aggressiveness and poor prognosis of the disease. In addition, the early diagnosis of glioblastoma is often tricky due to the lack of specificity of early clinical symptoms and the lack of early diagnostic tools. With the improvement of microsurgery technology, the application of various new radiotherapy techniques, and the introduction of chemotherapy and targeted drugs, the clinical prognosis of glioma has been improved to some extent. However, the overall survival rate of patients is still extremely poor, with a median survival time of only about 1 year, and almost all patients tend to relapse (Jhanwar-Uniyal et al., 2015; Kim et al., 2019). Therefore, developing a validated risk assessment model is essential to guide early clinical diagnosis, prognostic assessment, and individualized treatment.

Sarcopenia is an age-related syndrome of progressive decline in skeletal muscle mass and function, commonly seen in patients with malignancies and associated with poor prognosis in cancer patients (Williams et al., 2021). Several studies have shown that temporalis muscle thickness can be used as a surrogate marker for sarcopenia (Huq et al., 2021). Temporalis muscle thickness was also significantly associated with the expected survival of treated glioma patients with recurrent or concomitant brain metastases (Muglia et al., 2021). Quantifying temporalis muscle thickness has clinical value in the prognostic assessment of glioma (Guven et al., 2021; Mi et al., 2022). This study obtained co-expressed differential miRNA genes by analyzing glioma and skeletal muscle miRNA datasets, followed by multifactorial Cox regression analysis and stepwise regression analysis to obtain a patient survival risk regression model, which included FOSL1 and EN2.

FOSL1 belongs to the Fos gene family and encodes FOS-associated antigen 1 (FRA1), which is involved in forming a transcription factor complex AP-1 (Matsuo et al., 2000). In addition, as a proto-oncogene, FOSL1 also plays a vital role in tumorigenesis and can promote tumor cell metastasis through epithelial–mesenchymal transition (EMT). FOS1 also has prognostic value in various epithelial tumors, and its overexpression is associated with tumor aggressiveness, chemotherapy resistance, tumor progression, and poor survival prognosis (Yu et al., 2013; Gao et al., 2017; Vallejo et al., 2017; Xu et al., 2017; Sobolev et al., 2022). It has been shown that FOSL1 can promote the development and invasion of colorectal cancer through the Smurf1-mediated FBXL2/Wnt/β-catenin axis and the migration, invasion, and proliferation of breast, head, and neck squamous cell carcinoma, pancreatic cancer, bladder cancer, and prostate cancer (Elangovan et al., 2018; Luo et al., 2018; Cui et al., 2020; Dai et al., 2021; Hyakusoku et al., 2021; Liu et al., 2021). In addition, FOSL1 also mediates the dephosphorylation of proliferation and apoptosis bridging protein 15 (PEA15) by upregulating dual-specificity phosphatase 7 (DUSP7), which increases drug resistance in breast cancer (Li et al., 2022b). It is to be noted that FOSL1 was also associated with glioma growth and invasion and was a poor prognostic factor for GBM (Guo et al., 2022). Gliomas overexpress FRA1, which regulates the malignancy of gliomas, including morphology, growth pattern, and tumorigenic potential (Debinski and Gibo, 2005). The engrailed-2 (EN2) gene encodes a transcription factor containing a homology cassette involved in regionalization, patterning, and cellular differentiation in early brain development. It plays an essential role during nervous system development. In addition, disorders of EN2 regulation can lead to abnormal cell proliferation, leading to tumorigenesis. Studies show that EN2 plays an important role in the proliferation, migration, and invasion of various tumor cells, including prostate cancer, colorectal cancer, esophageal squamous cell carcinoma, lung cancer, and bladder cancer, and is closely related to the poor prognosis of tumor patients (Zhou et al., 2017; Lin et al., 2018; Cao et al., 2020; Li et al., 2020; Li et al., 2021). EN2 promotes the proliferation and invasion of colorectal cancer cells by regulating CCL20, thus promoting the progression of colorectal cancer. EN2 also promotes the invasion and metastasis of esophageal squamous cell carcinoma by upregulating SPARC expression. It promotes the proliferation, invasion, and metastasis of bladder cancer cells by activating the PI3K/Akt pathway and inhibiting the PTEN gene (Cao et al., 2020; Li et al., 2020) (Li et al., 2021). In addition, it was found that EN2 expression levels are also correlated with the degree of malignancy of gliomas and promoted the malignant progression of glioma (Zeng et al., 2020). These lines of evidence indicate that FOSL1 and EN2, which are involved in our risk assessment model, have prognostic value in various tumors, including glioma.

MiR-33a plays a variety of physiological roles in tumor microenvironments, and it has been proposed as a potential target for cancer prevention and therapy (Gao et al., 2020). By inhibiting phosphorylation of JAK2 and STAT3, which are highly activated in many malignant cells, including glioma cells, miR-33a inhibits the growth, invasion, and EMT of tumor cells (Feng et al., 2016; Chang et al., 2017; Liu et al., 2019a; Liu et al., 2019b). Aside from targeting PDE8A and UVRAG, miR-33a also targets cAMP/PKA and NOTCH signaling pathways in glioma cancer cells (Wang et al., 2014). It is to be noted that both signaling pathways promote self-renewal of glioma-initiating cells only when they are activated simultaneously (Jiang et al., 2019). MiR-33a may exert oncogenic effects by regulating JAK2/STAT3, cAMP/PKA, and NOTCH signaling pathways in gliomas. Further investigation is needed to determine whether miR-33a overexpression affects glioma cell proliferation, invasion, and other oncogenic features.

In this study, hsa-miR-33a was found to regulate FOSL1 and EN2 and affect the prognosis of gliomas. Furthermore, hsa-miR-33a appeared to be involved in sarcopenia and gliomas. However, a larger sample of data is needed to validate the findings conclusively, and further experimental studies are needed to help understand the specific regulatory mechanisms. Because this study only has data on skeletal muscle age, sarcopenia cannot be fully simulated. Furthermore, miR-33a downregulation caused by glioma and muscle aging may also be caused by sarcopenia, but this remains unclear as we did not investigate which causes miR-33a downregulation.

## Conclusion

In conclusion, this study’s risk model provides good clinical application and predictive value for assessing the risk and prognosis of glioblastoma and a potential therapeutic target. Moreover, hsa-mir-33a co-targeting FOSL1 and EN2 has a good predictive value for glioma and skeletal muscle reduction.

## Data Availability

The original contributions presented in the study are publicly available. This data can be found here: https://www.ncbi.nlm.nih.gov/geo/ GSE122488; GSE23527.Transcriptomic FPKM expression data was accessed from the UCSC Xena website (https://xenabrowser.net/).
